# Retinoic Acid Receptors in Acute Myeloid Leukemia Therapy

**DOI:** 10.3390/cancers11121915

**Published:** 2019-12-01

**Authors:** Orsola di Martino, John S. Welch

**Affiliations:** Department of Internal Medicine, Washington University, St Louis, MO 63110, USA; orsoladimartino@wustl.edu

**Keywords:** retinoid therapy, APL, AML, ATRA

## Abstract

Retinoic acid (RA) signaling pathways regulate fundamental biological processes, such as cell proliferation, development, differentiation, and apoptosis. Retinoid receptors (RARs and RXRs) are ligand-dependent transcription factors. All-trans retinoic acid (ATRA) is the principal endogenous ligand for the retinoic acid receptor alpha (RARA) and is produced by the enzymatic oxidation of dietary vitamin A, whose deficiency is associated with several pathological conditions. Differentiation therapy using ATRA revolutionized the outcome of acute promyelocytic leukemia (APL), although attempts to replicate these results in other cancer types have been met with more modest results. A better knowledge of RA signaling in different leukemia contexts is required to improve initial designs. Here, we will review the RA signaling pathway in normal and malignant hematopoiesis, and will discuss the advantages and the limitations related to retinoid therapy in acute myeloid leukemia.

## 1. Introduction

Nuclear receptors (NRs) are ligand-dependent transcription factors that bind DNA sequence-specific motifs in enhancers and promoters to transactivate their target genes [1]. The NR superfamily is characterized by a highly conserved structure consisting of a DNA-binding domain (DBD), a ligand-binding domain (LBD), and N and C-terminal transactivation domains. NRs can act as monomers, homodimers, or heterodimers [1,2,3].

The retinoic acid receptors (RARs) and retinoid X receptors (RXRs) are ligand-activated NRs that have pleiotropic effects including the control of hematopoietic stem cell self-renewal and differentiation [4,5,6]. There are three different isoforms of each receptor (α, β, and γ) that are differently expressed in mouse and human tissues [7,8].

Retinoid receptors are activated by retinoids, small molecules derived from dietary vitamin A. Vitamin A is a fat-soluble compound derived from beta-carotene found in plants and retinyl esters from animal sources. Following ingestion, vitamin A is stored in the liver and adipose tissue as retinyl esters and circulates in the blood in a protein complex containing retinol-binding protein and transthyretin [4,9]. Retinyl esters are reversibly oxidized to retinal by retinol dehydrogenase enzymes (ADHs) [10]. Retinal is irreversibly oxidized to all-trans retinoic acid (ATRA) by aldehyde dehydrogenases (ALDHs) [10,11]. In humans, 19 different ALDHs have been identified. Only a small proportion of circulating retinol is converted to ATRA (0.2–5%), and ATRA is subsequently further metabolized to inactive forms by cytochrome P450 enzymes (mainly CYP26 family) [12]. ATRA is considered the principal ligand for the retinoic acid receptor alpha (RARA) [13].

Retinoic acid (RA) signaling pathways regulate essential biological functions, such as cell development, differentiation, proliferation, and apoptosis, and the expression of retinoid receptors is downregulated in several tumor types [4,10,14,15]. RA signaling is crucial for mammalian post-implantation development and organogenesis, and vitamin A deficiency is associated with embryo and fetal malformation [15,16]. The availability of RA modulates the expression of genes involved in differentiation, and the RA gradient distribution thereby tunes the patterning of hematopoietic, hepatic, and neural stem/progenitor cell populations [4,6,16,17]. In the adult, RA signaling remains relevant in hematopoietic stem cells, where RARG activity plays a critical role in maintaining the stem cell pool [17,18].

RARs function as obligate heterodimers with RXRs. Ligand binding induces structural changes that alter the transcriptional activity of the heterodimer. In the absence of ligands, RAR-RXR acts as a transcriptional repressor by binding co-repressor complexes and recruiting histone deacetylases (HDACs) [19,20]. Ligand binding alters the position of helix 12, displacing the co-repressors and facilitating binding of co-activator complexes with histone acetylase activity (HATs). 

Retinoids have found a clinical role in the treatment of acute promyelocytic leukemia (APL) (M3 subtype of acute myeloid leukemia), which is associated with translocations involving *RARA* and which undergo maturation in response to ATRA [21,22]. APL is characterized by the pathognomonic presence of the fusion protein PML/RARA, which acts as a transcriptional repressor impairing the expression of genes that are critical to myeloid differentiation [23,24,25]. Retinoid therapy transformed response and survival outcomes of APL. Lo Coco et al. demonstrated that a combination of ATRA and arsenic trioxide (ATO) leads to complete remission (97% two-year event-free survival rates in the ATRA/ATO treated patients and *p* < 0.001) [25]. However, differentiation therapy with ATRA in non-APL acute myeloid leukemia (AML) has yielded mixed results, suggesting that subgroups of patients may possess greater or lesser retinoid sensitivity [26]. In particular, AML with nucleophosmin (*NPM1*) mutations [27,28], EVI-1 expression [29], and *IDH2* mutations [30] have been suggested to possess greater ATRA sensitivity. Additional studies examined the sensitivity to the RXR-selective ligand bexarotene, which also induces maturation and apoptosis in some AML cell lines and primary AML patient samples [31,32,33], suggesting a potential clinical role for other retinoids in cancer therapy.

## 2. Retinoid Acid Receptors: Structure and Mechanism of Action

RARs and RXRs have a conserved modular structure with an N-terminal ligand-independent activation function (AF-1), a central conserved DNA-binding domain (DBD), and a C-terminal ligand-binding domain (LBD) [34,35]. The multifunctional LBD is responsible for ligand binding and dimerization and contains a ligand-dependent activation function (AF-2), which corresponds to coregulator interaction surfaces that can be modulated by natural (e.g., retinoic acid) or pharmacological ligands (e.g., tamibarotene and bexarotene) [20,36,37]. RARs function as obligate heterodimers with RXRs, whereas RXR is a promiscuous heterodimerization partner with different nuclear receptors (e.g., peroxisome proliferator-activated receptors (PPARs), liver X receptor (LXRs), nuclear bile receptor (FXR), the thyroid hormone receptor (TR), and the vitamin D receptor (VDR)) [1]. 

The transcriptional activity of the retinoic acid receptor (RAR)-retinoid X receptor (RXR) heterodimer is regulated by the absence/presence of a binding ligand that generates conformational changes modulating the RAR-RXR complex [1,38,39]. In general, RAR-RXR dimers bind DNA with high affinity at specific retinoic acid response elements (RAREs) in target gene promoters/enhancers [19,40]. In the absence of a ligand (or in the presence of an antagonist), local transcriptional activity is repressed through the recruitment of the corepressor complexes (CoRs) in the promoter region of target genes [19,20]. The most common corepressors to interact with RAR:RXR heterodimers are the nuclear receptor corepressor (N-CoR) [41] and the silencing mediator for retinoid and thyroid hormone receptors (SMRT) [42], which are each capable of further recruiting histone deacetylases (HDACs) [40,43,44]. Local histone deacetylation then facilitates chromatin condensation and gene silencing [40]. In contrast, when an active ligand binds, this induces a structural shift in the C-terminal region of the LBD, helix H12, leading to destabilization of the CoR-binding and subsequent coactivator (CoAs) recruitment. The structural crosstalk between the RAR and RXR H12 regions is crucial for RAR regulation. Once bound, the CoA p160 family (TIF-2/SRC-1/RAC3) recruits histone acetyltransferase complexes (HATs) [45,46,47], which facilitate chromatin de-condensation and gene transcription activation. 

The corepressor N-CoR contains evolutionary conserved structured regions involved in transient intramolecular contacts. In the presence of RXR/RAR, N-CoR exploits its multi-valency to form a cooperative multisite complex that displays an equilibrium between different conformational states. Structural analysis of the RAR/RXR heterodimer revealed that in the absence of a ligand, the H12 helices are inclined to an extended helical position, whereas the presence of a specific ligand or receptor mutation results in re-orientation of this helix. This equilibrium is crucial to maintaining the repressive basal state while allows for the conversion to a transcriptionally active form [20]. A negative feedback mechanism controls RARA levels—upon ligand binding, RARA is ubiquitinated and degraded via the proteasome [14].

Different RXR heterodimers display different responsiveness to activation of individual elements in the diad. Some heterodimers function “permissively”, being capable of responding when the ligand binds either element of the heterodimer (e.g., PPAR/RXR, LXR/RXR, FXR/RXR) or “non-permissively”, being only capable of responding to ligands bound to the non-RXR element in the heterodimer (e.g., RAR/RXR, VDR/RXR and TR/RXR) heterodimers [1,48,49,50]. Steric interactions between the conformations of the two helices, 12 domains appear critical to modulate these phenotypes.

### 2.1. Retinoid Acid Signaling Pathway 

RA signaling controls the transcriptional activity of genes involved in cell growth, differentiation, and apoptosis in normal and cancer tissues. RARs and RXRs are expressed at early developmental stages in vertebrate embryos of various species, and RA signaling is fundamental for the maintenance of homeostatic control during embryonic and fetal development [17,51]. All-trans retinoic acid (ATRA) is the principal activator of RA signaling through binding to RARA receptors [52]. Upon uptake, Vitamin A is stored in the liver as retinyl esters [4]. In the plasma, retinyl acetate and retinol are largely protein-bound and can be found at micromolar concentrations [4]. Intracellular transport is regulated by retinol transporters such as STRA6 [53]. Once intracellular, retinol is oxidized by retinol dehydrogenase (RDH/ADH) to retinal, and then retinal is irreversibly oxidized to ATRA via retinal dehydrogenases (ALDH1A1, ALDH1A2, ALDH1A3, and Aldh3b1) [52,54]. The physiological levels of ATRA are further regulated through ATRA metabolism by cytochrome P450 enzymes (mainly, CYP26 family) [52]. 

ATRA is a highly potent agonist of all three RARs, with low nM EC50. In contrast, ATRA activates RXRs at near micromolar concentrations [4]. Further metabolism leads to active isomers such as 9-cis retinoic acid and 13-cis retinoic acid [4]. 9-cis retinoic acid has been identified as a potent RXR agonist, although the physiological relevance of this molecule *in vivo* remains controversial [55]. 13-cis retinoic acid is a less potent RARA ligand than ATRA [56,57]. 

Alternative, non-retinoic acid compounds have emerged as potential natural RXR ligands, specifically, long-chain fatty acids (e.g., docosahexaenoic acid (DHA) and C24:5) have been shown to be low-affinity RXR endogenous agonists and appear to be present in vivo at physiologically relevant concentrations [57,58]. 

#### 2.1.1. Vitamin A Deficient (VAD) Mice

**Figure 1:**
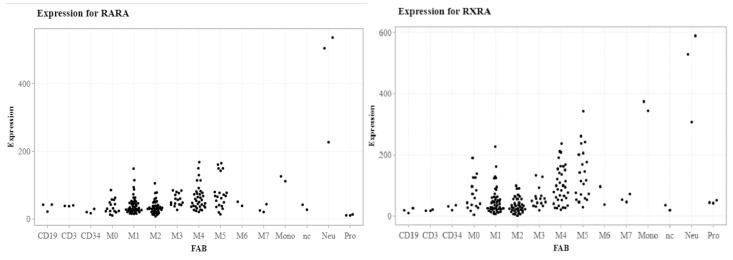
Expression profile of retinoic acid receptor alpha(RARA) and retinoic X receptor alpha (RXRA) in human samples. Expression profile of retinoid receptors in total bone marrow cells from 197 human AML cases and sorted normal bone marrow cells using RNA Seq. FAB is indicated on X-axis. (https://leylab.shinyapps.io/TCGA_AML_Web_App/).

RA signaling is essential for hematopoietic and immune system development, function, and homeostasis. In normal hematopoiesis, *RARA* and *RXRA* are dynamically regulated during myeloid maturation, with the highest expression in mature neutrophils (Figure 1) [41]. The function of natural retinoids has been explored by characterizing hematopoietic and immune phenotypes in Vitamin A deficient (VAD) mice. VAD mice display an abnormal expansion of myeloid cells (mainly terminally differentiating granulocytes expressing the Mac-1 and Gr-1 surface antigen) in bone marrow, spleen, and peripheral blood that can be reversed upon ATRA administration [59], whereas RARA dominant-negative mutations and RARA antagonists (i.e., BMS493 Pan-RAR inverse agonist) block myeloid maturation, leading to increased numbers of myeloid precursors [27,60,61]. Adult mice fed with a Vitamin A free diet for 14–17 weeks showed a reduction in bone marrow cellularity and a decrease of hematopoietic stem cells frequency and plasticity [18]. 

Although *RARA* and *RXRA* are expressed at lower levels in bone marrow B and T cells compared with neutrophils (Figure 1), VAD mice displayed compromised T and B cell immune-response phenotypes [59,60]. In B cells, VAD mice displayed altered proliferation and differentiation with a reduction of IgA isotype switching and reduced generation of antibody-secreting plasma cells [61,62]. VAD mice also showed an altered lymph node development with decreased T cell differentiation and reduced migration into tissues [61,62,63]. 

#### 2.1.2. Rars and Rxrs Knockout Mice

The contribution of each RAR and RXR isoform to hematopoiesis has been explored through the generation of knockout mice. During development, *Rara* is widely expressed, whereas *Rarb* and *Rarg* display tissue-specific expression patterns [64]. *Rara, Rarb,* and *Rarg* single knockout mice largely maintain normal hematopoiesis, suggesting that the different isoforms can play compensatory roles. The exception is *Rarg* knockout mice, which display a decreased number of hematopoietic stem cells (HSCs) and more mature progenitors [51]. *Rara/Rarg* double knockout mice show early lethality due to several embryonic malformations [6,65]. Fetal liver cells of *Rara/Rarg* double knockout mice are composed mainly of mature granulocytes similar to wild-type mice with no increase of *Rarb* expression level, suggesting that expression of *Rarb* is not modulated by the retinoid receptor feedback mechanisms [6]. 

RXRs are also expressed differentially in mouse tissues. *Rxra* and *Rxrb* have wide expression, whereas *Rxrg* is expressed in skeletal muscle and CNS, with limited (if any) expression in hematopoietic cells [39]. RXRs have been implicated in hematopoietic cell fate. RXRA downregulation is required for neutrophil terminal differentiation from human myeloid progenitors [39,66]. However, *Rxra* and *Rxrb* single knockout mice do not display gross defects related to hematopoiesis, supporting a compensatory role of these RXR isoforms [66,67]. *Rxra/Rxrb* double knockout mice show early developmental lethality due to the lack of formation of the labyrinthine zone of the chorioallantoic placenta [68]. 

### 2.2. Retinoid Acid Signaling Pathway in Cancer and Leukemia

RA signaling alteration has been implicated in the oncogenesis of diverse cancer types. Retinoic acid signaling has been implicated both as a tumor suppressor and an oncogene in a context-dependent manner. Enzymes involved in the chemical conversion of Vitamin A to ATRA are frequently down-regulated during oncogenesis [14]. Retinol acetyl-transferase downregulation has been associated with breast and skin carcinogenesis [10]. Cellular retinol binding protein 1 (CRBP1) is inactivated by gene-hypermethylation in human breast cancer [15,69], whereas the enzyme CYP26A1, that degrades the ATRA, is overexpressed [70,71]. Retinaldehyde dehydrogenase 1 (ALDH1) is a functional marker commonly used to identify cancer stem cells and its expression is associated with poor prognosis in several cancer types [72]. In prostate cancer, retinaldehyde dehydrogenase 2 (ALDH1A2) is silenced by locus hypermethylation and acts as a tumor suppressor [64,73]. Retinoic acid receptor beta 2 (*RARB2*) has been shown to have tumor suppressor activity in a series of human carcinomas (e.g., breast and prostate cancer) [74,75]. However, inactivation of stromal *Rarb* in the mouse results in a protective effect against ErbB2-induced mammary gland tumorigenesis [76], suggesting pleiotropic and tissue-specific effects. In a mouse model of chronic lymphocytic leukemia (CLL), characterized by the abnormal expansion of CD5+ B-cells in bone marrow and secondary lymphoid organs, there is increased RA signaling activity by leukemic cells with accumulation of ATRA in the stromal microenvironment as result. The inhibition of RA signaling with the pan-RARA inverse agonist, BMS493, reduced the leukemia burden of CLL cells in vitro and in vivo [77].

Recurrent *RXRA* mutations are found in 5–8% of human bladder cancer [78]. Mutant RXRA protein results in increased binding to the peroxisome proliferator activator receptors (PPARs) and hyperactivation of PPAR target genes that support tumor growth, raising the potential for therapeutic intervention targeting the PPARG:RXRA heterodimer [79].

Retinoid receptors exert transcriptional influence through coactivator and corepressor complexes. Across a range of cancer types, these proteins have also been observed to be dysregulated and to contribute to oncogenesis. The corepressors N-CoR and SMRT bind the androgen receptor (AR) and the estradiol receptor (ER), and their downregulation has been associated with breast and prostate cancer initiation, progression, and drug resistance [80]. In t(8;21) AML, AML1-ETO binds the corepressors N-CoR/SMRT and HDACs. Upon interaction with ETO, the N-CoR/SMRT/HDAC complex contributes to repression of genes that regulate hematopoietic precursors and terminal myeloid differentiation [81,82] 

Regulation of HAT and HDAC enzymes is crucial for gene transcription. HATs are among the most frequently mutated genes in urothelial carcinomas and upregulation of HDACs has been noted in a wide variety of cancers [83,84]. In particular, several HDACs have been found to be upregulated in acute lymphoblastic leukemia (ALL) and are associated with a poor prognosis. 5-azacytidine and 5-aza-2′-deoxycytidine (decitabine, DAC), a DNMT inhibitor, are used in the treatment in elderly patients with myelodysplastic syndromes (MDS) and AML [85]. Combinations of decitabine and HDAC inhibitors appeared logical, with pre-clinical synergistic effects on gene expression re-activation [86], but resulted in limited clinical efficacy in clinical trials [87].

#### 2.2.1. Retinoid Therapy in Acute Promyelocytic Leukemia

Acute Myeloid leukemia (AML) is the most frequent form of leukemia among adults, characterized by an uncontrolled accumulation of myeloid precursors cells. Ninety-five percent of acute promyelocytic leukemia cases are associated with the t(15;17)(q22:q11) chromosomal translocation, which results in the fusion of the promyelocytic leukemia gene (*PML*) and the *RARA* gene, generating the fusion protein “PML-RARA” [24,25]. Additional *RARA* fusion partners have also been described, although these occur with less frequency—the promyelocytic leukemia zinc finger (*PLZF*), nuclear mitotic apparatus (*NUMA*), and the signal transducer and activator of transcription 5b (*STAT5B*) or nucleophosmin 1 (*NPM1*) are the most common of these alternative partners [88]. Rare cases of *RARG* fusions have also been described with promyelocytic morphology [89]. The balanced translocation of t(15;17);t(17;15) is associated with the expression of a reciprocal fusion product (*RARA-PML*), and is accompanied by haploinsufficiency for both *PML* and *RARA* [90,91]. PML-RARA binds the HDAC complex with higher affinity than the RARA-RXR heterodimer, causing a block of differentiation at the promyelocytic stage due to gene transcription repression [92]. PML-RARA forms homodimers and can heterodimerize with wild type PML and RXR, inhibiting their functions.

**Figure 2:**
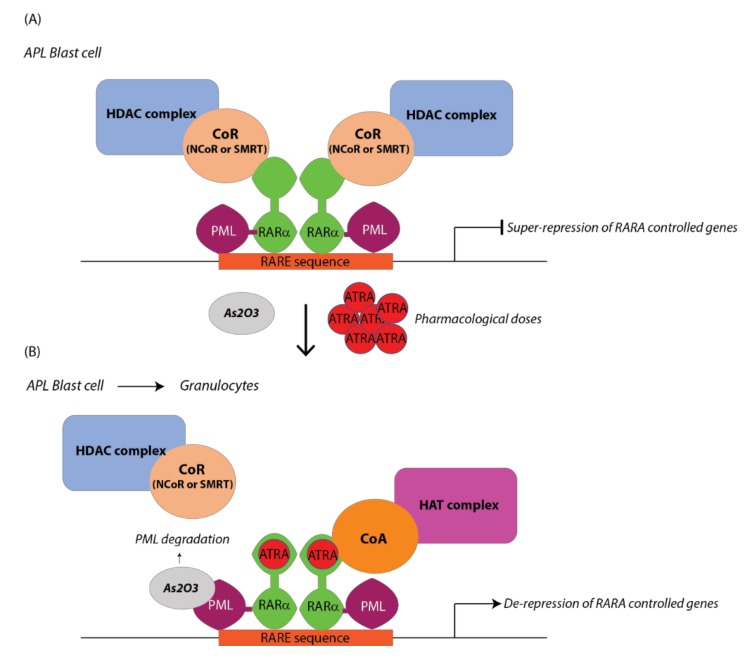
Differentiation therapy in APL. (**A**) In APL blast cells the fusion protein PML-RARA forms oligodimers and binds the DNA with higher affinity than the wild type RARA. The binding of PML-RARA to RARA sequence allows the co-repressor (CoR)-HDAC complex recruitment leading to gene expression repression. (**B**) Administration of pharmacological doses of ATRA leads to CoR-HDAC release and recruitment of the co-activator (CoA)-HAT complex that activates gene expression.

APL is an aggressive form of AML, however, combination therapy with ATRA and arsenic trioxide (ATO) is highly effective [25,93]. Pharmacologic doses of ATRA induce the displacement of the HDAC complex from the fusion protein PML-RARA and unlock the terminal differentiation of APL blasts to granulocytes (Figure 2). ATRA binding leads to subsequent PML-RARA degradation [94]. In parallel, ATO binds to PML, leading to its degradation in parallel with APL blast differentiation and apoptosis [25,95]. 

#### 2.2.2. Resistance to Differentiation Therapy in APL Patients

A recurrent cause of acquired clinical resistance to ATRA treatment in APL patients is the acquisition of missense mutations (e.g., Leu290Val, Arg394Trp, and Met413Thr) in the RARA-LBD of the fusion protein, PML-RARA [14]. This form of resistance emerges most commonly at the second relapse, and in patients treated with low-dose consolidation or maintenance chemotherapy [96]. Non-PML-RARA translocations tend to be resistant to ATRA/ATO. PLFZ-RARA is able to bind and transactivate its target gene with higher affinity than PML-RARA. A second resistance mechanism involves overexpression of cellular retinoic acid-binding protein (CRABP1), which may reduce intracellular free ATRA availability [97]. PLFZ is a transcription factor and can regulate the recruitment of CoA even in the presence of pharmacological concentrations of ATRA. The molecular mechanisms underlying ATRA-resistance in STAT5b-RARA patients are not fully understood. One of the reasons could be related to the presence of STAT5b-RARA in the nucleus (Stat5b is localized in cytoplasm) with subsequent aberrant expression of both RARA and STAT5b target genes. Neither PLZF-RARA nor STAT5b-PML patients appear sensitive to ATO, likely because ATO effects occur through its binding to PML [98,99].

The synthetic retinoid tamibarotene (Am80) has been tested in APL patients to overcome ATRA resistance in high-risk patients. Tamibarotene is ten times more potent than ATRA, has binding specificity for RARA, and has low affinity for CRABP1 [97]. In addition, it is more stable to light, heat and oxidation than ATRA, and its plasma levels do not decline after daily administration [100]. Tamibarotene was approved in Japan in 2004 for APL treatment but is not available in the United States. Results from a Japanese clinical trial (JALSG-APL204) with a median follow-up of 7.3 years showed the relapse-free survival (RFS) at 7 years was 93% in the tamibarotene arm and 84% in the ATRA arm (*p* = 0.027, HR = 0.44, 95% CI, 0.21 to 0.93) [101].

#### 2.2.3. Retinoid Acid Signaling Pathway as a Potential Target for Acute Myeloid Leukemia Therapy

Differentiation therapy with ATRA in non-APL AML has yielded mixed results [26]. Therefore, subgroups have been sought to identify patients with enhanced retinoid sensitivity. AML is associated with altered expression of chromatin modifying genes, such as the histone deacetylase HDACs, the DNA methyltransferase DMNTs, and the mono- and di-methyl lysine demethylase LSD1 (KDM1A) [102]. Cases with high LSD1 expression have been associated with epigenetic suppression of *RARa2* expression [102,103]. Consistent with this, treatment with the LSD1 inhibitor, trans-2-phenylcyclopropylamine (TCP), augmented retinoic acid differentiation and ATRA-sensitivity in ATRA responsive and non-responsive AML cell lines [104]. 

Super-enhancer analysis in primary AML samples identified a sub-group of AML patients with a super-enhancer in the *RARA* locus, which are highly responsive to SY-1425, a potent and selective RARA agonist [105]. 

Patients with *NPM1* mutations may also be preferentially sensitive to ATRA or to combinations of ATRA and ATO. Subgroup analysis suggested that the addition of ATRA to chemotherapy was associated with improved responses in *NPM1* mutated-AML patients. These results were supported by the in vitro sensitivity of NPM1 mutated-AML cells treated with ATRA/ATO, which induced proteasome-mediated apoptosis [27,28,106].

AML with *IDH2* mutations have been associated with *LSD1* de-regulation and a transcriptional signature of ATRA sensitization. Consistent with this, the ATRA/arsenic trioxide combination led to responses in model systems with phenotypes suggestive of the differentiation syndrome [30]. Finally, EV1-1 enhanced expression occurred in almost 10% of AML patients and is correlated with poor outcomes (overall survival of <1 year). Intriguingly, ATRA treatment lead to blasts differentiation and reduction in leukemic engraftment in a subset of patients-derived primary human AML cells with ecotropic viral integration site 1 (EVI-1) overexpression [29,107]. 

Finally, ATRA may serve as a chemosensitizer. ATRA increases the expression of CD38, a transmembrane glycoprotein expressed on hematopoietic cells and the target of Daratumumab. Pre-clinical data suggests this combination augments leukemia cell death [108].

#### 2.2.4. Role of Autophagy in Acute Myeloid Leukemia Retinoid Therapy

Autophagy is a key mechanism that maintains cellular metabolism and homeostasis through degradation of intracellular components, such as organelles and damaged or redundant proteins [109]. The autophagosome is a bi-membrane vesicle that envelops the cellular content targeted for degradation. This highly conserved mechanism protects cells from metabolic/environmental stress (e.g., nutrient deprivation, hypoxia, and chemotherapies) and controls cell self-renewal, differentiation, and death [110]. Autophagy is crucial for the maintenance of HSCs. Autophagy gene expression levels are downregulated in APL cells resulting in a low autophagy activity, which may cooperate with PML-RARA to lead to the development of leukemia [111]. It has been shown that ATRA administration can stimulate autophagosomes accumulation and induce degradation in APL and non-APL cell lines by stimulating granulocytes differentiation [112]. 

#### 2.2.5. Rexinoids in Acute Myeloid Leukemia Therapy

Rexinoids are a class of potent synthetic specific RXR ligands, which have been explored in AML [39,113,114]. Bexarotene is a pan-RXR ligand that is approved by Food and Drug Administration agency in the United States for the treatment of cutaneous T-cell lymphoma (CTCL) and has been tested for other cancer types [113]. Several studies have been conducted to show the efficacy of bexarotene in AML. An initial study of relapsed or refractory AML treated with bexarotene as monotherapy results in the induction of myeloid differentiation in two patients [115]. Differentiation induced by bexarotene was also noted in AML cell lines, and the pro-differentiative effect could be enhanced by the combination with the LXR agonist T0901317 [33]. A second clinical trial combined increased bexarotene doses with decitabine (DNA hypomethylation agent) in older and relapsed AML patients. This combination was well tolerated in patients but also resulted in only modest responses [116]. 

## 3. Conclusions

Natural and synthetic derived retinoids are a class of compounds commonly used as therapeutic agents in oncology. Retinoids have been explored in diverse cancer types, including lung cancer, breast cancer, and AML, and are FDA approved to treat APL and cutaneous T cell lymphoma. In the case of APL, the combination of ATRA and ATO has resulted in striking efficacy and long term survival. Retinoid treatments in other leukemia subtypes have resulted in mixed outcomes, although emerging biomarkers suggest that subgroups may exist with enhanced sensitivity. Further understanding of the co-activator/co-repressor interactions with the retinoid receptors may enable broader application of retinoids in diverse cancer types.
